# Archaeal LOV domains from Lake Diamante: first functional characterization of a halo-adapted photoreceptor

**DOI:** 10.3389/fmicb.2025.1572269

**Published:** 2025-06-13

**Authors:** Lorena Valle, Yonathan J. Coronel, Guillermina E. Bravo, Joaquín A. Díaz, Virginia Helena Albarracín, María E. Farías, Inés Abatedaga

**Affiliations:** ^1^Molecular Photobiology Laboratory, Instituto de Bionanotecnologia del NOA (INBIONATEC), CONICET, Santiago del Estero, Argentina; ^2^Facultad de Agronomía y Agroindustrias (FAyA), Universidad Nacional de Santiago del Estero (UNSE), Santiago del Estero, Argentina; ^3^Laboratorio de Microbiología Ultraestructural y Molecular, Centro Integral de Microscopía Electrónica (CIME), CONICET-Universidad Nacional de Tucumán, Camino de Sirga s/n, Finca El Manantial, San Miguel de Tucumán, Argentina; ^4^Facultad de Ciencias Naturales e Instituto Miguel Lillo, Universidad Nacional de Tucumán, San Miguel de Tucumán, Argentina; ^5^PUNABIO S.A. Campus USP-T Av. Solano Vera y Camino a Villa Nougués San Pablo, San Miguel de Tucumán, Argentina

**Keywords:** LOV photoreceptor, Archaea, flavoprotein, extremophiles, halo-adapted protein

## Abstract

High-altitude Andean lakes (HAALs) represent polyextreme environments where diverse photoinduced processes have been documented. In this study, we investigated Light-Oxygen-Voltage (LOV) photoreceptors and identified 28 archaeal sequences from Lake Diamante, which were classified into two major groups (A and B), with three outliers showing unique structural features. Analysis of these sequences and their 3D models revealed hallmark adaptations to halophilic environments, including an abundance of surface acidic residues, an increased prevalence of arginine over lysine, and a greater density of salt bridges. The heterologous expression of a representative LOV domain, ALovD-1, demonstrated conserved photophysics between its dark- and light-adapted states, which was consistent with the slow cycling type. Importantly, ALovD-1 exhibited remarkable halophilic characteristics, maintaining photocycling functionality at salt concentrations as high as 3 M monovalent salts. This ability can be attributed to discrete structural changes, allowing adjustments in flavin interactions within its cavity under varying ionic strengths. Mutational studies of key residues (Y30F and Y48F) highlighted their roles in modulating flavin photophysic and revealed a stabilizing function for Y48 at low salt concentrations. These findings mark the first functional characterization of a canonical archaeal LOV domain, expanding our understanding of light sensing and protein adaptation in extremophiles.

## 1 Introduction

Photosensing in microorganisms living in extreme environments has been extensively studied, offering valuable insights into the molecular mechanisms that enable survival under harsh conditions. High-Altitude Andean Lakes (HAALs), located in the Andean Puna region of South America, present a unique opportunity to explore such mechanisms. These lakes, often considered polyextreme environments, combine high UV radiation, low oxygen levels, elevated pH, and high salinity, creating a natural laboratory for studying extremophiles (Albarracín et al., 2016; Alonso-Reyes and Farias, 2020). Microbiomes in these habitats, particularly in microbialites and stromatolites, exhibit diverse photoinduced molecular adaptations. For example, microbial rhodopsins and photolyases isolated from these environments are critical components of the cellular machinery adapted to extreme conditions, contributing to UV resistance and light energy utilization (Alonso-Reyes et al., 2022; Albarracín et al., 2014; Toneatti et al., 2017; Portero et al., 2019; Gorriti et al., 2023).

Among photoresponsive proteins, Light-Oxygen-Voltage (LOV) domains, a subfamily of the Per-Arnt-Sim (PAS) fold, are particularly notable for their involvement in light sensing across Bacteria, Eukarya, and, to a lesser extent, Archaea. LOV domains, such as those from *Bacillus subtilis* YtvA, *Avena sativa* LOV2, and *Pseudomonas putida* PpSB1-LOV, have been extensively characterized in model systems, revealing the mechanisms underlying their photochemical behavior (Taylor and Zhulin, 1999; Salomon et al., 2000; Krauss et al., 2005; Losi et al., 2002; Schwerdtfeger and Linden, 2003). Similarly, LOV domains have been identified in the microbialites of Socompa Lake, which are predominantly affiliated with Cyanobacteria and aerobic anoxygenic phototrophic bacteria (Alonso-Reyes et al., 2022). However, knowledge about archaeal LOV domains is limited, as most studies have focused on metagenomic surveys without detailed functional characterization. Thus, the functional and structural properties of archaeal LOV domains remain largely unexplored (Herrou CS, 2011; Rascovan et al., 2016; Losi et al., 2014; Pathak et al., 2009).

Lake Diamante, located within the Galán Volcano crater at an altitude of approximately 4,600 m, exemplifies a polyextreme environment. Owing to its high pH (Salomon et al., 2000; Krauss et al., 2005; Losi et al., 2002), elevated salinity, and arsenic concentrations, as well as low oxygen levels, the lake mimics ancient Earth conditions, making it an exceptional site for studying microbial adaptation and evolution. The microbial communities in the lake, dominated by Archaea from the Euryarchaeota phylum, exhibit remarkable adaptations, including arsenic respiration and a prevalence of mobile genetic elements associated with resistance mechanisms (Rascovan et al., 2016; Ordoñez et al., 2018; Sancho-Tomás et al., 2020; Perez et al., 2020). A metagenomic database was generated from the red biofilms that were found at the bottom of calcareous rocks (microbialites) in Lake Diamante. Their taxonomical analysis revealed that they were mostly composed of microorganisms from Archaea (Euryarchaeota phylum) (Rascovan et al., 2016). Preliminary analysis highlights the presence of LOV domains in these datasets affiliated with Euryarcheota. However, their functional roles and adaptations to extreme conditions have not been systematically studied.

Extreme environments impose remarkable evolutive pressure on microorganisms that attempt to adapt to survive. The Halophile Archaea overcomes hypersalinity by actively pumping Na^+^ ions out of their cytoplasm to the exterior of the cell, which results in a relative concentration of K^+^ ions at the intracellular level, with a final concentration of 4–5 M needed to reach an isosmotic level with the environment (Christian and Waltho, 1962). This favors the presence of proteins that have an increased number of acidic residues (glutamate and aspartate) on the surface of the protein that interact with Na^+^ and K^+^ ions, which helps stabilize the structure and reduces the overall hydrophobic content (Aharon, 2013). Halophilic microorganisms have acidic proteomes (Paul et al., 2008).

Thus, in this work, we analyzed the occurrence and taxonomy of LOV domains in the metagenome of Lake Diamante and studied their primary amino acid structure. Phylogenetic analysis revealed that all the sequences obtained were of archaeal origin and that they were predominantly distributed in two groups, with some minor outliers. The similarities and differences in the primary and tertiary structures of one selected sequence, named Archaeal LOV Domain-1 (ALovD-1), with those of its mesophilic counterparts are presented here. Spectroscopic characterization of ALovD-1 revealed that the photophysics of the dark and light-adapted states were fairly conserved with respect to LOV domains from other species. The photocycling capacity of this domain was tested with increasing monovalent salt concentrations, proving that it remains functional at KCl concentrations equivalent to those found in haloarchaeal cytoplasms. These findings offer new insights into light sensing and protein adaptation mechanisms in extremophilic Archaea, with potential implications for biotechnology and evolutionary biology.

## 2 Materials and methods

### 2.1 Selection of LOV domains from Lake Diamante

BLAST searches were performed in the Integrated microbial genomes and microbiomes (IMG/M) database (Chen et al., 2023) using the deposited metagenomic dataset obtained from the red biofilm microbial communities from calcareous rocks in Lake Diamante (IMG_3300011121). *Bacillus subtilis* YtvA and *Avena sativa* LOV2 were used as queries. The settings were an e value of 10^—07^, and all sequences were further screened for the superconserved motif in LOV domains (GXNCRFLQG) and 14 other conserved residues (Pathak et al., 2009). Only two sequences were found with a proline instead of the superconserved cysteine, and two others were found with tyrosine instead of phenylalanine in the conserved motif. Some hits consisted of incomplete open reading frames (ORFs), and their full-length counterparts were blasted against the NCBI database (BLASTP) to obtain the best match to assign taxonomy. The conserved domains database (NCBI) was used to identify the protein architecture using the Pfam database. Analysis of the sequences consisted of alignment of the LOV domains only (ca. 120 residues) and was performed via MUSCLE (Madeira et al., 2024) with default parameters and Jalview (Waterhouse et al., 2009) for visualization. From the alignment, a phylogenetic tree was built via PhyML 3.0 (Lefort and Longueville, 2017). All interactions among residues were calculated via RING4.0 (Del Conte et al., 2024).

### 2.2 Construction of the expression plasmid and protein production and purification

The Ga0151614_185661 LOV domain (372 bp, Supplementary Figure S2) and the mutants Y30F and Y48F were synthesized by GenScript (United States). They were subcloned and inserted into the pET28a+ plasmid via the restriction sites NcoI and XhoI without a stop codon to produce C-terminal His-tagged fusions, namely, ALovD-1, Y30F and Y48F. These plasmids were expressed in BL21 (DE3) pLysS cells and induced with 0.5 mM isopropyl-*β*-d-thiogalactopyranoside (IPTG) at 14°C for 16 h in the dark in terrific broth (TB) media. Protein was purified using 20 mM Tris/HCl pH 8, 0.5 M NaCl as lysis buffer and Ni-NTA agarose (Invitrogen). The fractions were eluted with a gradient (0–250 mM imidazole) and then analyzed using SDS-PAGE according to the methods of Schägger (2006). Those with pure protein were desalted prior to use in buffer containing 10 mM PBS, pH 8, and 0.5 M NaCl at 8°C until use or as indicated elsewhere.

### 2.3 Spectroscopic measurements

Absorption spectra were recorded as previously reported (Abatedaga et al., 2017; Golic et al., 2019) using a modular miniature UV–vis spectrophotometer USB2000 + (Ocean Optics, United States) connected via an optic fiber to a UV–vis light source (Analytical Instrument System, United States). A total of 250 μL of fresh ALovD-1 protein solution, in a 5 × 5 mm quartz cuvette (Hellma, Mulheim, Germany) was fixed in a cuvette holder and connected to a circulating fluid bath (Lauda T) for temperature control. Second derivative spectra were applied to the ultraviolet region of the absorption spectrum (200–300 nm) to study peak shifts of tyrosine and tryptophan in the presence of cations (K^+^ and Na^+^). They were calculated using a nine-point data filter and third-order Savitzky–Golay mathematical differentiate, using Origin 8.5 software from Microcal™. A spline function was applied to the resulting spectra, and interpolated points were used to reach 0.01–0.02 nm resolution. This analysis was applied to ALovD-1 WT spectra in 10 mM PBS, pH 8, supplemented with 0.5, 1, 2 and 3 M NaCl or KCl. The absorption spectra were screened for scattering and changes under UV light to avoid changes in concentration due to protein aggregation or denaturation at all times.

Steady-state fluorescence emission spectra were recorded with a Hitachi F-2500 spectrofluorometer (Kyoto, Japan) equipped with a Hamamatsu R-928 photomultiplier. Emission spectra of ALovD-1 were obtained by selective excitation of the FMN cofactor at 450 nm. The light intensity was attenuated by a 50% neutral density filter. The FMN fluorescence quantum yield Φ_F_ in the ALovD-1_446_ state was calculated by chemical actinometry using FMN in a water solution [Φ_F_ = 0.26 (Holzer et al., 2002)]. The cofactor anisotropy r was determined as previously reported (Villegas et al., 2014) via the classical L-format and calculated with Equation 1, where I_VV_ and I_VH_ are the fluorescence intensities with different orientations of the excitation and emission polarizers (Melles Griot, United States). The subscripts V (vertical) and H (horizontal) indicate the polarizer positions. The G factor represents the sensitivity ratio of the detection system for vertically and horizontally polarized light, which is calculated as I_HV_/I_HH_.


(1) $$r=\frac{I_{\text{VV}}-G\;I_{\text{VH}}}{I_{\text{VV}}+2\;G\;I_{\text{VH}}}$$

All measurements were registered at 15°C in ALovD-1 solution; 10 mM PBS, pH 8; and 0.5, 1, 2 and 3 M NaCl or KCl, as indicated in the corresponding section.

The fluorescence dynamics of ALovD1_446_ were determined in 10 mM PBS pH 8, 0.5 M NaCl. Lifetimes and time-resolved anisotropy were registered using a Tempro-01 time-correlated single photon counting (TCSPC) system (Horiba, Glasgow, United Kingdom), and the data were analyzed as described elsewhere (Abatedaga et al., 2017). Briefly, the fluorescence intensity decays obtained at 500 nm and 520 nm (emission bandwidths of 8 nm) by excitation with a pulsed blue-LED (Nanoled 461 ± 27 nm, 1 MHz, Horiba) were fitted with the fluorescence decay analysis software DAS6 (Horiba) by deconvoluting the pulse function using a multiexponential model function, Equation 2:


(2) $$I_{\left(t\right)}=\sum_{i=1}^{n}\alpha_{i}\;(-t/\tau_{i})$$

(3) τav=∑i=1nfiτi=∑i=1nαiτi2∑i=1nαiτi

(4) $$r_{t}=r_{o}\exp\;(-t/\theta )$$

where n is the number of single exponential decays and where τ_i_ and α_i_ are the decay time and the fluorescence intensity amplitude at t = 0 of each decay, respectively. The average lifetime (τ_av_) was calculated with Equation 3, where *f*_i_ is the fractional contribution of each decay time to the steady-state intensity.

The fluorescence anisotropy decays were analyzed with the classical exponential model function for a spherical emitter, Equation 4, where r_0_ is the maximum anisotropy at t = 0 and *θ* is the rotational correlation time of the sphere. The temperature control in the fluorescence experiments was performed using a circulating fluid bath (Haake F3) connected to the cuvette holder.

All experiments were performed in triplicate, and the average values with standard deviations are reported.

### 2.4 Photoactivation quantum yield of ALovD-1 photoproduct formation

The light-adapted state for ALovD-1 (ALovD-1_390_) was obtained by blue light irradiation of the dark-adapted form (ALovD-1_446_) using a 1 W Royal Blue LED (Luxeon Star Leds) at 443 ± 20 nm until a steady state was reached, as determined by absorption absorbance (plateau). The photoactivation or photoadduct formation quantum yield of ALovD-1_390_ (Φ_for_) was estimated following a methodology previously described (Abatedaga et al., 2017), adapted to ALovD-1_390_ formation (Equation 5). The CALovD-_390_ concentration was determined from the slope of the decay kinetic profile at 446 nm. Δε = 7,640 M^−1^ cm^−1^ was calculated from ε_FMN_ at 450 nm in ALovD1_446_ = 14,200 M^−1^ cm-^1^ (see below), and the normalized absorption spectra at the isosbestic point of the ALovD-1_446_ and ALovD-1_390_ absorption spectra were obtained. The photon flux, q_np_, was determined by chemical actinometry using a potassium ferrioxalate salt solution.


(5) Φfor=CALovD−1390qnp

The extinction coefficient of FMN in ALovD-1_446_ was determined according to Wingen et al. (2014). The absorption spectra of the proteins were recorded at 15°C, after which the samples were incubated at 90°C for 10 min and finally centrifuged at 10,000 rpm for 15 min. The absorption spectra of the supernatant, with the chromophore dissociated from the protein, were recorded again. The ratio of both absorption values was then multiplied by the extinction coefficient of free FMN 12,200 M^−1^ cm^−1^ at 450 nm (Whitby, 1953). The thermal recovery percentages (%_rec_) were calculated via Equation 6, where *Abs_t0_* is the absorbance value at 466 nm at t_0_ and *Abs_ti_* is the absorbance value at t_i_ (3 or 16 h).


(6) $${\%}_{\text{rec}}=\frac{\text{Ab}s_{\text{ti}}}{\text{Ab}s_{t0}}\;x\;100\;$$

All experiments were performed in triplicate, and the average values with standard deviations are reported.

### 2.5 Modeling and structural analysis

Sequences for proteins ALovD-1 (WT, Y30F and Y48F mutants), 6,091-AI and 0912-AII were modeled using Alphafold2 (Mirdita et al., 2022). Models were visualized using Chimera (Pettersen et al., 2004) and PyMOL (Schrödinger, 2010). Residue interactions were detected using RING4.0 with default parameters (Clementel et al., 2022). Surface-exposed residues were detected using SwissPDB viewer with a cutoff of ≥10% surface accessibility and GETAREA (Fraczkiewicz and Braun, 1998).

## 3 Results

### 3.1 LOV domains from Lake Diamante are of archaeal origin

**Figure 1:**
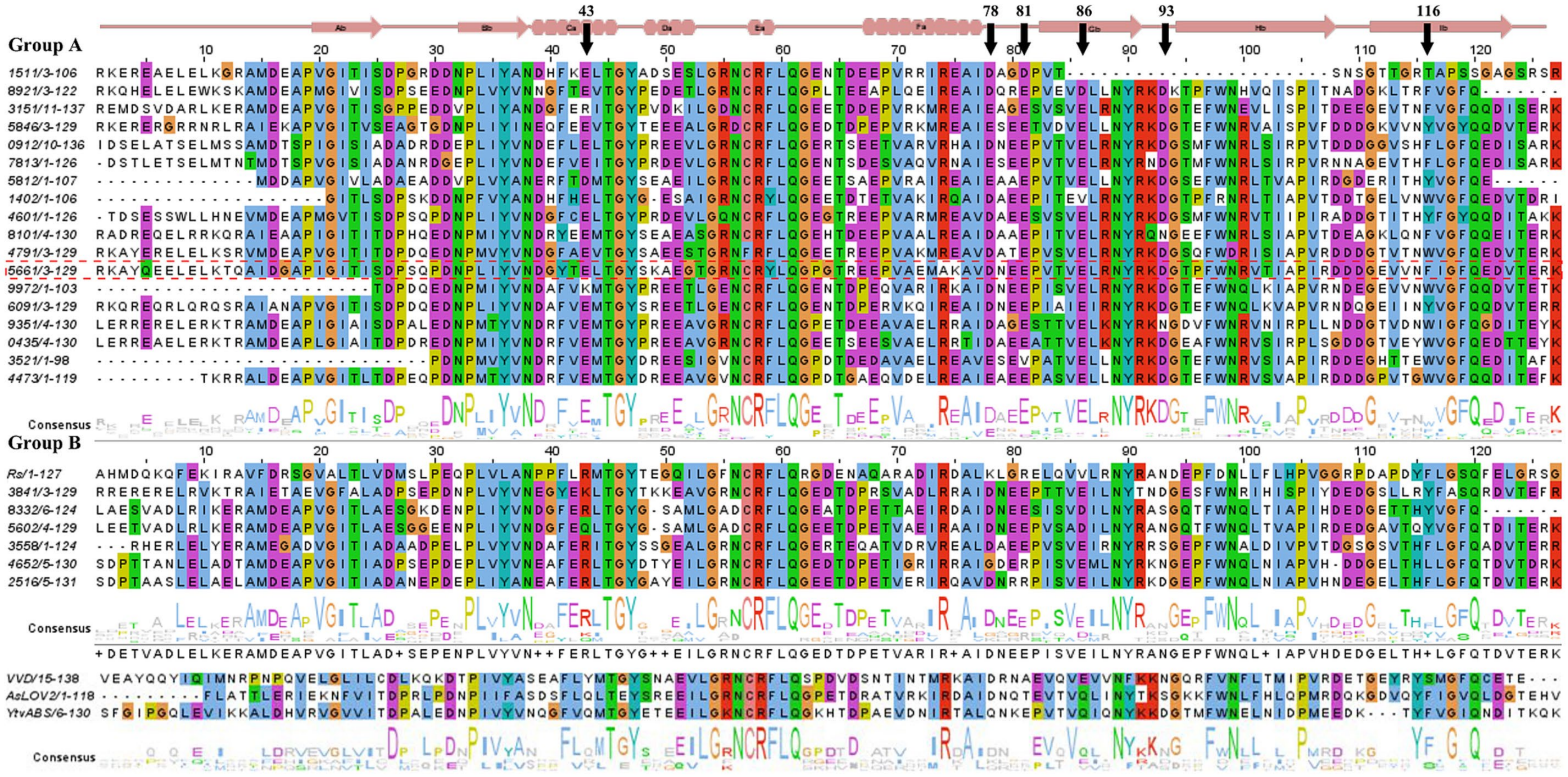
Sequence alignment of Diamante Lake-LOV with selected photoactive LOV domains (YtvA, VVD, *As*LOV2 and *Rs*LOV). The secondary structural features are represented at the top of the figure. The residue numbering is arbitrary. The arrows indicate residues that are referred to in the text. Diamante Lake-LOV sequences are grouped on the basis of the phylogenetic analysis from Figure 2, i.e., groups A and B. Sequence identities are the last 4 numbers from the annotations in the metagenome of the selected gene ORFs (refer to Supplementary Table S1).

All residue numbers from the archaeal sequences found in Lake Diamante in this section are shown in Figure 1.

Exploration of red biofilm mats from the Lake Diamante metagenome database revealed 28 sequences encoding putative LOV domains. BLAST against nonredundant protein sequences at the National Center for Biotechnology Information (NCBI) databases allowed us to assign the probable taxonomical origin for each candidate sequence and to account for those sequences that were incomplete in the metagenome assembly (i.e., incomplete contigs, Supplementary Table S1). All of them were from the Archaea domain, with 30%, 63%, and 7% of the sequences corresponding to the Halobacteriales, Haloferacales and Natrialbales orders, respectively. The percentages of the metagenomic sequences containing the LOV domain (complete or incomplete ORFs) and the best match found in the NCBI nonredundant protein database ranged from 47%–93%, with the exception of a 32% identity assigned to *Salarchaeum* sp. JOR-1 (Supplementary Table S1).

**Figure 2:**
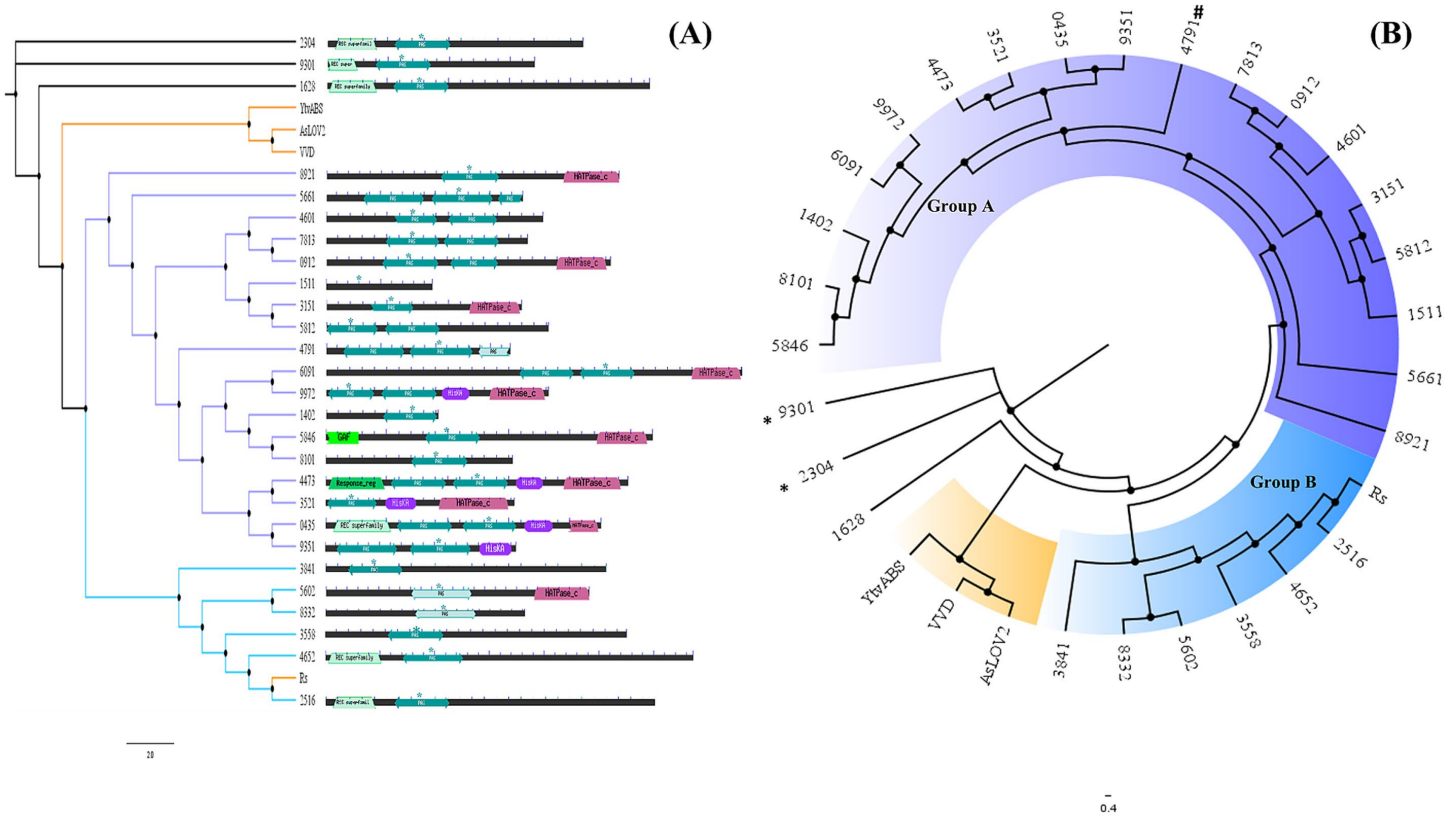
**(A)** Neighbor-joining tree generated with Muscle alignment and default parameters combined with domain architecture as detected by CDD via the Pfam database. Diamante Lake-LOV sequences were used as found in the metagenome assembly; in some cases, they were incomplete. **(B)** Cladogram representation. The sequences with * correspond to those with a proline residue in the LOV motif instead of cysteine and (#) with a phenylalanine.

Analysis of each sequence via a conserved domain database (CDD) search against the Pfam database revealed that most LOV domains (characterized as PAS domains) were part of complex architectures (Supplementary Table S2). They generally involve more than one sensor module in the N-terminus (response_regulator from the REC superfamily, PAS and GAF domains), and at the C-terminus, there are mostly histidine kinases, as shown in Figure 2A, as described previously (Losi et al., 2014).

A phylogenetic tree performed using only the LOV domain sequences (ca. 120 residues) from Diamante grouped 24 of these archaeal sequences into two major clusters (A and B), except for 3 LOV domains that did not group even with the reference sequences included in the analysis (YtvA, VVD and *As*LOV2), suggesting that differential archaeal lineages are present in this extreme environment (Figure 2B). Two of the ungrouped sequences have proline residues instead of the hyperconserved cysteine in the LOV motif (9,301 and 2,304). One other sequence grouped in cluster A has phenylalanine at the same position (4,791). As has been described, groups only share LOV-related structural similarity, and they do not represent putative molecular functions or taxonomical relationships, meaning that the grouping of LOV domains observed may be found only for this habitat (Taylor and Zhulin, 1999).

The alignment of all the sequences revealed differentially conserved residues in the archaeal LOVs compared with the sequences from Bacteria and Eukarya (Supplementary Figure S1). The most surprising, though not unexpected, hallmark for halo-adapted proteins found throughout most archaeal LOVs is an abundance of acidic residues (glutamate and aspartate), most commonly located in coils and toward the chromophore site of the antiparallel *β*-sheet. An example is loop Fα-Gβ (Figure 1), which has a conserved motif DXEE (residues 78–81). Similarly, E86 (located in Gβ) and Asp93 (in the Gβ-Hβ β-turn in group A), which are present separately in VVD and YtvA, respectively, are also conserved along all archaeal LOV domains, most likely helping in the stabilization of the protein surface.

Other interesting features found were, e.g., in position 116, in which published sequences from other domains of life have exclusively a phenylalanine residue. This residue is inside the chromophore site and is most likely in contact (through ππ stacking and van der Waals interactions) with the isoalloxazine ring itself. In archaeal LOVs from Diamante, this residue can be Trp or Tyr in most cases. These two aromatic compounds are known to be strong deactivating factors of the excited state of flavins, which leaves the question as to what role they might play in physiological processes.

The archaeal LOVs of Groups A and B differ. The alpha helices C, E and F strongly contained acidic residues. Group B sequences have an arginine at position 43, as opposed to a conserved glutamate in group A. Coil Eα-Fα, located immediately after the LOV motif, has a distinctive acidic residue composition compared with previously characterized LOV domains. β-sheets G and H have conserved features throughout the archaeal sequences compared with the reference sequences in Figure 1. For example, Gβ has a negatively charged residue at position 86 and an arginine at position 88 in group A only. In the case of Hβ, the hyperconserved FWN motif is followed by arginine or glutamine in groups A and B, respectively. Coil Gβ-Hβ (residues 91 to 94) in group A sequences is relatively conserved compared with the reference LOV sequences in the sense that the first two positions are positively charged residues and the last is a conserved glycine. In archaeal sequences, the first residue is invariably an arginine (instead of a lysine in all characterized LOV domains), and in group A, the third position is an aspartate.

### 3.2 ALovD-1, a putative LOV domain from *Halonotius* sp.

The sequence Ga0151614_185661 was selected for characterization. The ORF contains 360 amino acids (aa), with only 125 aa corresponding to a putative LOV domain, which was cloned and heterologously expressed in *E. coli* for subsequent purification. Ga0151614_185661 has 84.5% identity with accession WP_120100563 from *Halonotius aquaticus* (726 amino acids), implying that the original sequence obtained from the metagenome is incomplete (Supplementary Figure S2). *Halonotius* genus accounted for 4% of the amplicon-based 16S rRNA profiling in Lake Diamante (Rascovan et al., 2016). Ga0151614_185661 and its homologs encode a PAS domain containing a histidine kinase. The second PAS domain (of three PAS domains present in the full-length protein) contains conserved residues that are consistent with an LOV domain; therefore, it was chosen for heterologous expression and purification. ALovD-1 (from now on) belongs to group A of archaeal LOV sequences according to phylogeny (Figure 2B), although it is grouped separately from the rest of the sequences in the cluster and hence does not share all the conserved residues described in the previous section. Modeled ALovD-1 using Alphafold2 shows a conserved LOV arrangement (although not identical) at a secondary (Aβ, Bβ, Gβ, Hβ, and Iβ; and Cα, Dα, Eα, Fα and Jα) and tertiary structural level compared with the known LOV domains from prokaryotic and eukaryotic referents. The consensus GXNCRFLQ is present, and C46 is a strong candidate for forming the photoadduct. The LOV motif of ALovD-1 is different: the F48 position is a Tyr. An important characteristic of ALovD-1 regarding structural stability in halo-adapted proteins is the presence of 5–9 potential intrachain salt bridges on the protein surface, depending on the model used (Supplementary Figure S3), in contrast to the 1 and 4 found in YtvA (2MWG) and VVD (3RH8), respectively (Supplementary Table S3). They consist of one internal helical salt bridge (K38-D28), 3 internal helical bridges (E61-K64-E70 and K116-E119) and one helical bridge (E69-R96). The four remaining salt bridges are located in loops. To understand whether this number of ionic bridges was significant in other archaeal LOV sequences, two sequences from group A (DL6091 and DL0912, see Supplementary material) were modeled and then analyzed via RING 4.0. Ten and six ionic bridges were found in these proteins, respectively (Supplementary Table S3). Surface-exposed residues have also been used to identify adaptive characteristics in proteins from extremophile microorganisms. Supplementary Table S4 shows two evident tendencies for ALovD-1 (together with DL-6091, DL-0912 and reference sequences). The first is the high abundance of acidic residues, i.e., Glu and Asp., and the second is an increase in Arg with fewer Lys residues on the protein surface.

### 3.3 ALovD-1 is a *bona fide* photoreceptor that responds to blue light

**Figure 3:**
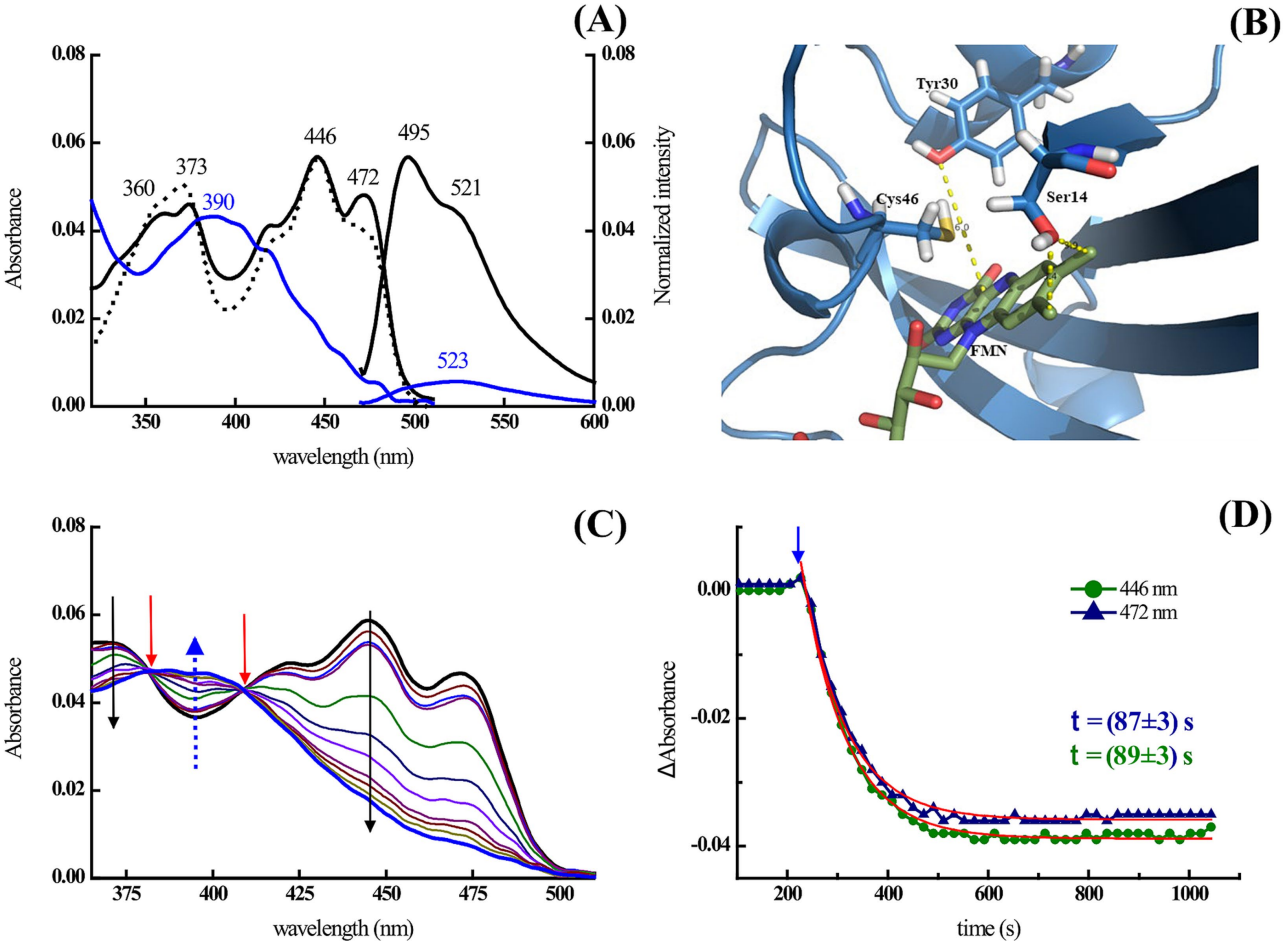
**(A)** Absorption and fluorescence emission spectra of ALovD-1_446_ (black) and ALovD-1_390_ (blue). Excitation spectra at 520 nm for ALovD-1_446_ (dotted line). **(B)** Chromophore binding site detail in the ALovD-1 model. Cys57 is involved in adduct formation, and the proximity of Ser14 to C7M and C8M and Tyr30 is possibly responsible for flavin deactivation. **(C)** Spectral changes of ALovD-1_446_ upon illumination with blue light (443 ± 20 nm). The black arrows indicate decreasing bands, and the blue arrows indicate the appearance of the photoproduct at 390 nm. Red arrows indicate isosbestic points at 380 and 409 nm. **(D)** Kinetic profile of ALovD-1_390_ formation, followed at 2 different wavelengths: 446 nm (green circles) and 472 nm (blue triangles), with arrows indicating initial illumination.

ALovD-1 was heterologously expressed and purified (Supplementary Figures S4A,B). Primary *in vitro* photophysical characterization was performed using PBS + 0.5 M NaCl. The purified protein was proven to have flavin mononucleotide (FMN) as a chromophore (Supplementary Figure S4C). Dark-adapted ALovD-1 absorbs with two maxima (Figure 3A). The band at 446 nm, which corresponds to the fully oxidized flavin, is accompanied by two shoulders at 422 and 472 nm, which are generally assigned to the vibronic state transition S_0_ → S_1_. The second maximum is in the UVA region and has a double peak at 350 and 370 nm, corresponding to an S_0_ → S_2_ transition. This double peak was attributed to the presence of a serine residue in the vicinity of the methyl substituents of the isoalloxazine ring (C7 and C8 of ring I). Modeled ALovD-1 confirms that Oᵧ from Ser14 is placed at 3.9 and 3.4 Ǻ to C7M and C8M of flavin, respectively, as shown in Figure 3B (Raffelberg et al., 2013). When the dark-adapted state (ALovD-1_446_, from now on) was illuminated with blue light, the formation of the light-adapted state of the protein was evidenced by the appearance of a maximum at 390 nm, as expected for a functional LOV domain and attributed to cysteinyl adduct formation (i.e., ALovD-1_390_). These details are summarized in Table 1.

**Table 1:** Absorption properties of ALovD-1 WT, Y30F and Y48F.

Protein	Condition	λ max abs	A_sh_/A_max_	τ_LOV390_ (s)	ε_446_ (M^−1^ cm^−1^)	Φ_for_	Recovery^c^
ALovD-1_446_ WT	0.5 M NaCl	446	0.82 ± 0.01^a^	89 ± 3	14,200	0.49 ± 0.07 ^a^	68/100
	1 M NaCl	446	0.87 ± 0.03^a^	87.75 ± 6	13,365 ± 800	0.45 ± 0.04 ^a^	73/100
	2 M NaCl	446	0.81 ± 0.02	77.25 ± 7	13,898 ± 70	0.54 ± 0.07 ^a^	60/100
	3 M NaCl	446	0.81 ± 0.01	75 ± 4,5	15,720 ± 1,040	0.4 ± 0.06	62/85
	0.5 M KCl	446	0.80 ± 0.02	99 ± 13	ND	0.57 ± 0.03^b^	72/100
	1 M KCl	446	0.82 ± 0.02^b^	79.5 ± 7	14,975 ± 390	0.57 ± 0.06^a,b^	86/100
	2 M KCl	446	0.81 ± 0.03^a^	93 ± 20	13,691 ± 650	0.36 ± 0.05^a,b^	63/100
	3 M KCl	446	0.84 ± 0.02^b^	88.7 ± 15	13,045 ± 1,105	0.45 ± 0.08	55/100
ALovD-1-Y30F_446_	0.5 M NaCl	446	0.77 ± 0.01^b^	95 ± 60	ND	0.53 ± 0.05	ND
ALovD-1-Y48F_446_	0.5 M NaCl	446	0.83 ± 0.07^b^	38 ± 2	ND	0.13 ± 0.01^b^	ND

Significance (*p*-value < 0.05): ^a^between present and next concentration, ^b^between same concentrations different salts. ^c^% rec_3hs_/% rec_ON_. τ_LOV390_: kinetics followed at 446 nm. Error: ± standard deviation.

The fluorescence emission of the flavin cofactor in ALovD-1_446_ has a maximum at 495 nm, with a prominent vibrational transition centered at 520 nm (Figure 3A). Rearrangement of the interactions between flavin and surrounding residues after the formation of ALovD-1_390_ is reflected in a drastic reduction in the emission, with a bathochromic effect on the maximum and a loss of structure resembling a free flavin. When the protein was returned to the dark, allowing thermal relaxation back to ALovD-1_446_, the emission recovered its structure, confirming that flavin was not released from the cavity (see Discussion below).

Kinetic studies were performed to determine the time of ALovD-1_390_ formation (Figure 3C) as well as for thermal recovery back to ALovD-1_446_ following the *Δ* absorbance at 446 and 472 nm. Monoexponential fitting of the photodepletion of the dark-adapted state resulted in *τ*_LOV390_ = 89 ± 3 s and 87 ± 3 s (Figure 3D) in PBS + 0.5 M NaCl at 446 and 472 nm, respectively. The thermal recovery time (τ_rec_) was evaluated after 3 h in the dark at 15°C; 68% of the protein reverted to ALovD-1_446_. The protein fully returned to the dark-adapted state when it was incubated overnight at 8°C.

Kinetic profiling allowed us to calculate the photoactivation quantum yield of ALovD-1_390_ or the yield of photoadduct formation, Φ_for_ = 0.49 ± 0.07 in PBS + 0.5 M NaCl. We have chosen Φ_for_ value in previous works to compare the formation of the thioadduct instead of τ, since it integrates all structural and local protein changes and, more importantly, is independent of protein concentration and instrumental conditions (Golic et al., 2019).

### 3.4 ALovD-1 remains functional at high salt concentrations

We used the photoactivation quantum yield (Φ_for_) of ALovD-1_390_ at several Na^+^ and K^+^ chloride salt concentrations to test whether high salt concentrations affect the functionality of the photoreceptor. We found that for both cations, the overall effect was a reduction of approximately 20% in the photoactivity at a concentration of 3 M compared with that at a 0.5 M salt concentration (Table 1). However, the behavior was different: the best performance of Φ_for_ for ALovD-1 for K^+^ was at 1 M, whereas that for Na^+^ was at 2 M (*p* value < 0.05). The proximity of aromatics to the flavin cofactor in its cavity, as measured by the A_sh_/A_max_ ratio, increased consistently and significantly with increasing salt concentration, suggesting that an aromatic residue in the vicinity of FMN interacted more closely under these conditions. In the case of K^+^, the maximum was reached at the highest concentration tested (3 M), whereas for Na^+^, this ratio reached a maximum at 1 M and then decreased to values similar to those at low salt concentrations (0.5 M). Therefore, from a functional point of view, ALovD-1 maintains its ability to photocycle at salt concentrations as high as 3 M of monovalent salts.

**Figure 4:**
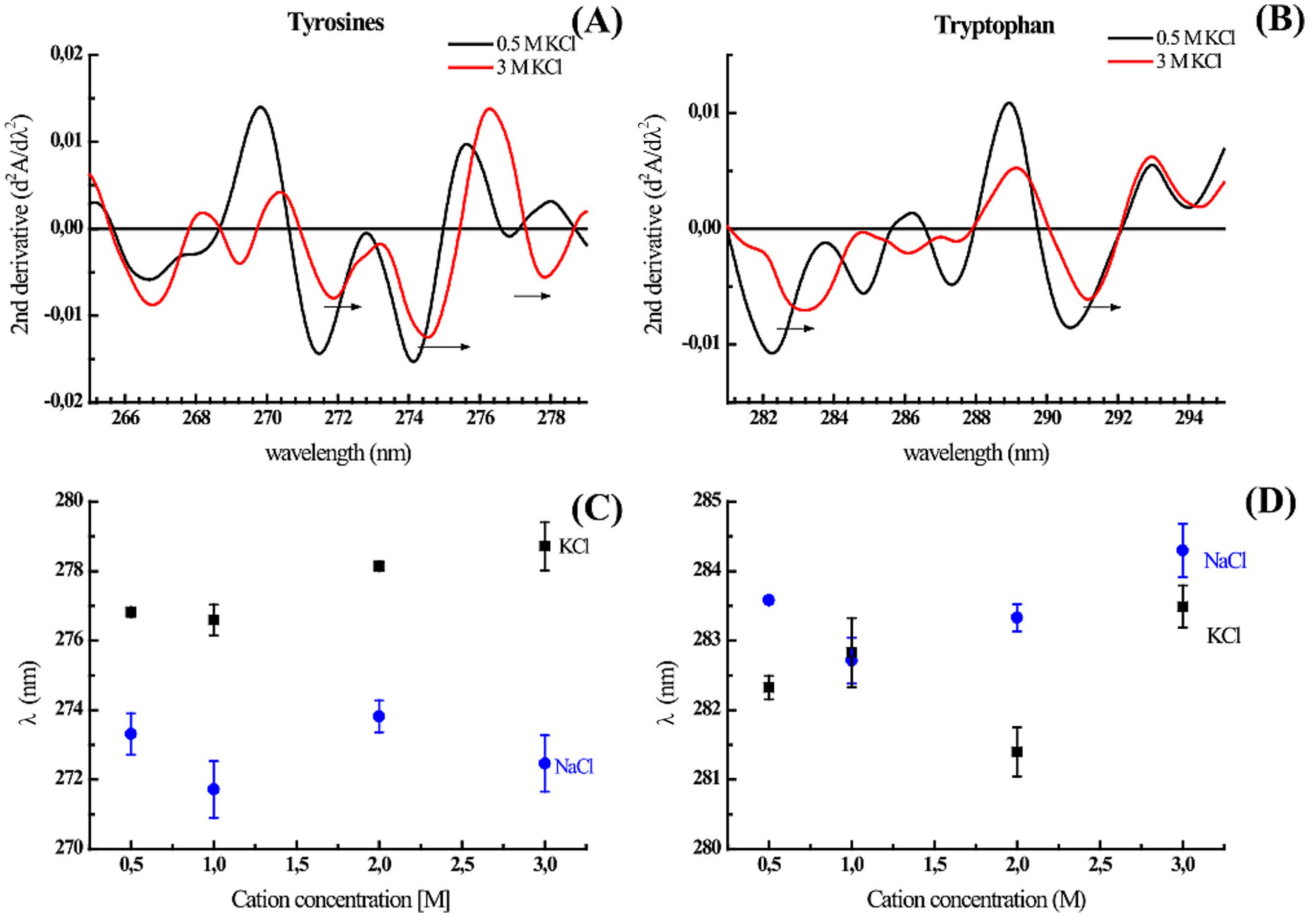
**(A,B)** Second derivate of UV absorption spectra of ALovD-1_446_ in PBS with 0.5 M KCl (black line) and 3 M KCl (red line). The arrows indicate a bathochromic shift at characteristic residue minima. **(C,D)** Tyrosine and tryptophan negative peaks at 277 nm and 283 nm, respectively, vs. the monovalent salts NaCl (black squares) and KCl (blue squares).

We wanted to explore whether some discrete rearrangements were present by analyzing the absorbance and fluorescence spectroscopy data. Although it is possible to calculate the solvent accessible surface area (SASA) for each residue (Supplementary Table S5), the change in the surface exposure of aromatic residues such as tryptophan and tyrosine when the protein structure is perturbed by high salt concentrations can be estimated/studied via different approaches. According to the second derivative spectra of the UV absorbance of the protein, the increase in K^+^ and Na^+^ concentrations resulted in a redshift of several minima associated with the contribution of tyrosine and tryptophan to the protein UV absorption spectrum (265–285 nm) (Figure 4). The 277 nm minimum is considered a distinctive mark of tyrosine, and the cation concentration progressively redshifts (Figures 4A–C); therefore, fewer cation–*π* interactions are induced by greater residue hydrophobicity (burial). The minimum at approximately 283 nm is representative of the Trp contribution in the presence of Na^+^ and K^+^ (Figures 4B–D). At 3 M of both salts, this minimum redshifted, suggesting that the only tryptophan in ALovD-1 was more buried than it was at all the other concentrations (*p* value < 0.05). Following the intrinsic fluorescence emission (Supplementary Figure S5), we find the same trend, which in this case represents a blueshift of the emission maxima, together with a small but significant decrease in the emission FWHM (Table 2). Taken together, the shielding of aromatic lateral chains from the protein surface toward the hydrophobic core when the protein is in high salt concentrations is the expected behavior, and these results confirm that this is the case for ALovD-1, while the photoreceptor remains active. Moreover, as reported elsewhere (Möglich, 2007), some residues seem to be more related to protein stability and are not necessarily involved in photophysical processes.

**Table 2:** Intrinsic fluorescence emission properties of ALovD-1.

Condition	λ_max_ emission	FWHM
0.5 N NaCl	342 ± 1	59
3 N NaCl	335 ± 1	57
0.5 N KCl	342 ± 0.6	59
3 N KCl	336 ± 0.1	56

### 3.5 Flavin fluorescence reveals details of the ALovD-1 binding site

The fluorescence quantum yield (Φ_F_) of the chromophore in ALovD-1_446_, upon excitation at 450 nm, was 0.14 ± 0.01 in PBS for FMN. Table 3 presents ALovD-1 average lifetimes (*τ*_av_), components (τ_i_) and their fractional contributions (% *f*_i_) for FMN. These parameters vary with the detection wavelength. The fluorescence decay for FMN in ALovD-1_446_ is complex and requires 3 components to fit it properly. At 500 nm, representing the emission maxima of flavin in ALovD-1, the very short component, τ_1_ = 50 ps, is the predominant population, with a fractional contribution of 52%, followed by the longest component (τ_3_ = 3.37 ns and *f*_3_ = 38.3%). Instead, when acquisition is taken at 520 nm, corresponding to the shoulder in the emission, the longest component becomes more abundant (τ_3_ = 3.4 ns and *f*_3_ = 62.6%) at the expense of the shortest component (τ_1_ = 0.10 ns and *f*_1_ = 20%). The variation in these parameters with wavelength may be interpreted as arising from two conformations of the protein-chromophore, each giving rise to 3 possible components. At 500 nm, the shortest component is more abundant, meaning that the rapidly deactivated state is predominant. In contrast, at 520 nm, the longest τ becomes predominant and may represent a more relaxed population. Free FMN in PBS was fitted satisfactorily with only two components, with the longest component surpassing the lifetime of the longest component in ALovD-1.

**Table 3:** Steady-state and dynamic fluorescence emission parameters of native ALovD-1_446_ and the mutants Y30F_446_ and Y48F_446_ after flavin chromophore excitation in PBS with 0.5 M NaCl.

Protein	Φ_F_	λ	<r>	τ_1_	τ_2_	τ_3_	τ_av_	*f_1_*	*f_2_*	*f_3_*	*χ* ^2^
ALovD-1	0.14	500 nm	0.28	0.05 ± 0.01^b^	1.2 ± 0.11	3.37 ± 0.05^b^	1.44 ± 0.5^b^	52 ± 16^b^	9.7 ± 1.9^b^	38.3 ± 14^b^	1.16
		520 nm	0.27	0.1 ± 0.01	1.2 ± 0.28	3.4 ± 0.07	2.36 ± 0.3	19.9 ± 11	17.5 ± 1	62.6 ± 11	1.11
Y30F	0.16^a^	500 nm		0.07 ± 0.003^a^	1.6 ± 0.02 ^a^	3.4 ± 0.05	1.02 ± 0.01	61.2 ± 0.3	19.1 ± 0.1^a^	19.7 ± 0.2^a^	1.32
		520 nm		0.05 ± 0.03^a^	1.5 ± 0.04 ^b^	3.7 ± 0.02^a,b^	1.8 ± 0.1^a,b^	30.2 ± 6^b^	37.4 ± 4.6^a,b^	32.1 ± 0.8^a,b^	1.25
Y48F	0.07^a^	500 nm		0.1 ± 0.01^a^	-	2.9 ± 0.06^a^	0.46 ± 0.09^a^	87.0 ± 4^a^	-	13 ± 3.9 ^a^	1.38
		520 nm		0.13 ± 0.03	-	2.9 ± 0.02^a^	1.64 ± 0.3^a,b^	45.5 ± 9^a,b^	-	54.5 ± 9.3^b^	1.21
FMN	0.26^a^	525 nm	0.03	0.34	-	4.57	4.43	3	-	97	1.16

Significance (*p* value < 0.05): ^a^between mutant and WT, ^b^between 500 and 520 nm for the same protein (WT or mutant). Error: ± standard deviation.

**Figure 5:**
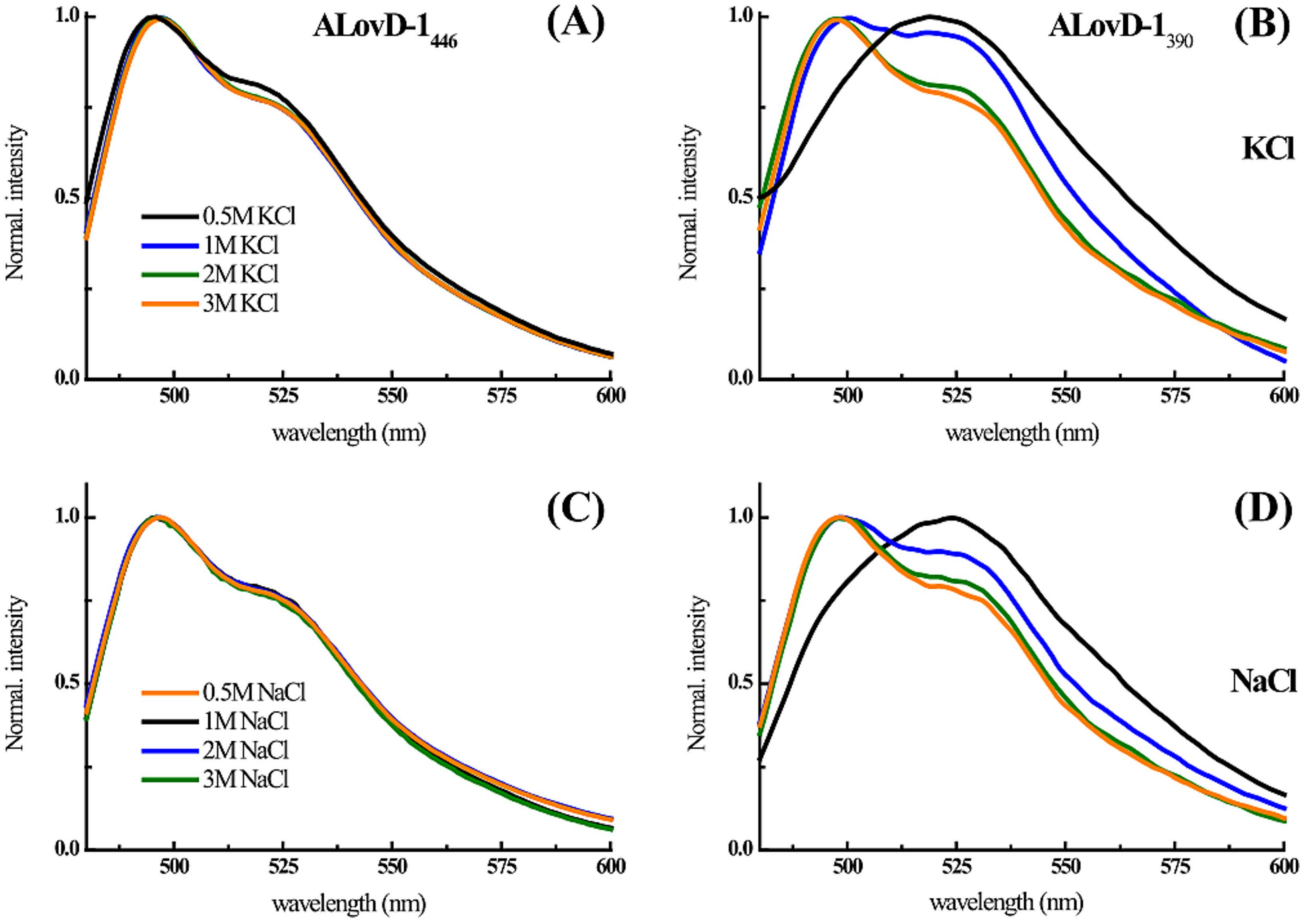
**(A–C)** ALovD-1_446_ and **(B–D)** ALovD-1_390_ normalized emission spectra in PBS with different KCl and NaCl concentrations: 0.5 M (black lines), 1 M (blue lines), 2 M (green lines) and 3 M (orange lines).

We also studied the steady-state fluorescence of FMN in ALovD-1 at increasing salt concentrations (Table 4). ALovD-1_446_ did not change with increasing salt concentration for either cation tested, with a conserved maximum and only a slightly more defined shoulder at 520 nm for concentrations of cations of 1 M or higher. However, for ALovD-1_390_, we noticed that for the initial low concentrations of Na^+^ and K^+^, there was no structure in the emission spectra, with the maxima shifting to 524 and 519 nm, respectively. These findings prompted the question of whether FMN was released from its interaction with the protein. First, we subjected ALovD-1 to sequential series of exposures to blue light and then recovered in the dark following the kinetics of photodepletion and thermal relaxation at 446 nm (Supplementary Figure S6). The protein maintained its activity, suggesting that the flavin at 0.5 M of these salts keeps its interaction with the residues surrounding it (highly conserved Q50, N78, N88 and Q109 through H-bonds) while sensing a more polar environment. Additionally, if the flavin was unbound from the protein, instead of a strong decrease in the intensity of the emission, an increase in the latter would be evidence of free flavin, which is not the case here (as will be commented upon later for the Y48F mutant). Then, ALovD-1_390_ flavin emission was analyzed for concentrations of 1 M or higher salt, and we found that the emission spectra gained structure with increasing salt concentration (for both Na^+^ and K^+^), as depicted in Figure 5. Hence, we speculate that there is a threshold salt concentration in the solvent that allows ALovD-1 to interact properly with FMN by keeping the solvent out of the flavin cavity such that the range of concentrations we tested is above 1 M. We also determined the Φ_F_ of ALovD-1_446_ with increasing Na^+^ and K^+^ concentrations (Table 4). For Na^+^, the Φ_F_ did not significantly change at any of the concentrations tested (*p* value > 0.05). For K^+^, ALovD-1 had a better yield at the lower concentration used, showing a very small but significant decrease when the salt concentration increased, but at 3 M, the Φ_F_ value was recovered at 0.5 M salt. Thus, ALovD-1 seems to be able to conserve its response to light with increasing ionic strength by adjusting its interaction with the flavin in its cavity and preserving the photophysics underlying photoadduct formation.

**Table 4:** Steady-state fluorescence properties of ALovD-1 WT at different salt concentrations.

Protein	Condition	λ _max emi_ & λ _sh emi_	FWHM (nm)	<r > (500 ± 5)nm	<r > (520 ± 5) nm	Φ_F_
ALovD-1_446_ WT	0.5 M NaCl	495/521	61	0.35 ± 0.01	0.34 ± 0.02	0.14 ± 0.01
	1 M NaCl	496/521	62^a^	0.38 ± 0.01	0.38 ± 0.001	0.14 ± 0.01
	2 M NaCl	496/521	60	0.37 ± 0.01	0.37 ± 0.02	0.14 ± 0.01
	3 M NaCl	497/521	60	0.37 ± 0.004	0.36 ± 0.01	0.15 ± 0.01
	0.5 M KCl	496/521	61	0.35 ± 0.02	0.33 ± 0.02	0.16 ± 0.01^a,b^
	1 M KCl	497/521	60^b^	0.34 ± 0.01^b^	0.33 ± 0.01^b^	0.15 ± 0.01
	2 M KCl	497/521	60	0.35 ± 0.02^a^	0.35 ± 0.02^a^	0.14 ± 0.01^a^
	3 M KCl	497/520	60	0.39 ± 0.03	0.39 ± 0.03	0.16 ± 0.01
FMN	water	525	75	0.01 ± 0.002	0.01 ± 0.002	0.26

Significance (*p* value < 0.05): ^a^between present and next concentration, ^b^between same concentrations of different salts. Error: ± standard deviation.

The rotational correlation time for the flavin in ALovD-1 also shows a dependence on the monitoring wavelength, with θ_500 nm_ = 1.06 ns and θ_520 nm_ = 2.00 ns. These values are quite low but differ from those of the free flavin in the buffer solution. However, these θ_ALovD-1_ values are not expected if they are calculated with a spherical rotor model via Perrin’s equation (θ = τ_av_ [(r_o_/r) − 1]^−1^), which would yield θ = 4.5 ns, which could be interpreted as rapid depolarization caused by flavin mobility in its cavity. In contrast, stationary anisotropy reveals that mobility is not a good option since <r > _500_ = 0.28 ± 0.01 and <r > _520_ = 0.27 ± 0.01, implying a moderate constraint compared with free FMN in solution, which results in <r > _FMN_ = 0.03 and θ_FMN_ = 0.86 ns (Supplementary Figure S7). Hence, the rapid depolarization observed must be caused by other factors that involve the protein–chromophore environment rather than mobility.

### 3.6 Role of Tyr30 and Tyr48 in FMN deactivation

The quantum yield of fluorescence (Φ_F_) of FMN in ALovD-1 is half that of FMN in water, which is well described in the literature for flavoproteins in general, since the surrounding residues in the protein cavity modulate the photophysics of flavin. It is also relatively low compared with other LOV domains, i.e., YtvA and CrLOV1 (Φ_F_ = 0.22 and 0.17, respectively) (Losi et al., 2002; Holzer et al., 2002), although ALovD-1 is in the same order as *As*LOV2, CrLOV2 and Mr4511 (Φ_F_ = 0.13, 0.12 and 0.14, respectively) (Holzer et al., 2005; Kawano et al., 2013; Consiglieri et al., 2020). The deactivation effect on flavin emission is also observable in the dynamic fluorescence emission parameters, as described in the previous section (Table 3). To test which residue might be responsible for this deactivation and on the basis of the predicted models of the protein, we first targeted Y30, with its OH at 6.15 Å, to flavin C4. The Y30F mutant was produced and purified (Supplementary Figure S4B). Its absorbance parameters are presented in Table 4, and it has a Φ_for_ = 0.53 and the ratio A_sh_/A_max_ is lower (0.77) than in the WT since the Tyr near the FMN has been mutated (Table 1). Compared with the WT protein, this mutant presented a small but significant increase in the Φ_F_ to 0.16. The fluorescence decay was fitted with a multiexponential function with 3 components, similar to that of the WT. Although τ_av500_ does not change, the two shortest lifetimes present a moderate but significant increase, whereas *f*_2_ increases at the expense of *f*_3_ at 500 nm. At 520 nm, the shortest component decreases significantly, whereas the largest component increases in lifetime. In this case, de τ_av520_ was strongly reduced compared with that of the WT. Therefore, in this context, eliminating a strong quencher, such as Tyr near flavin, had a moderate effect on FMN deactivation, contrary to what we expected.

**Figure 6:**
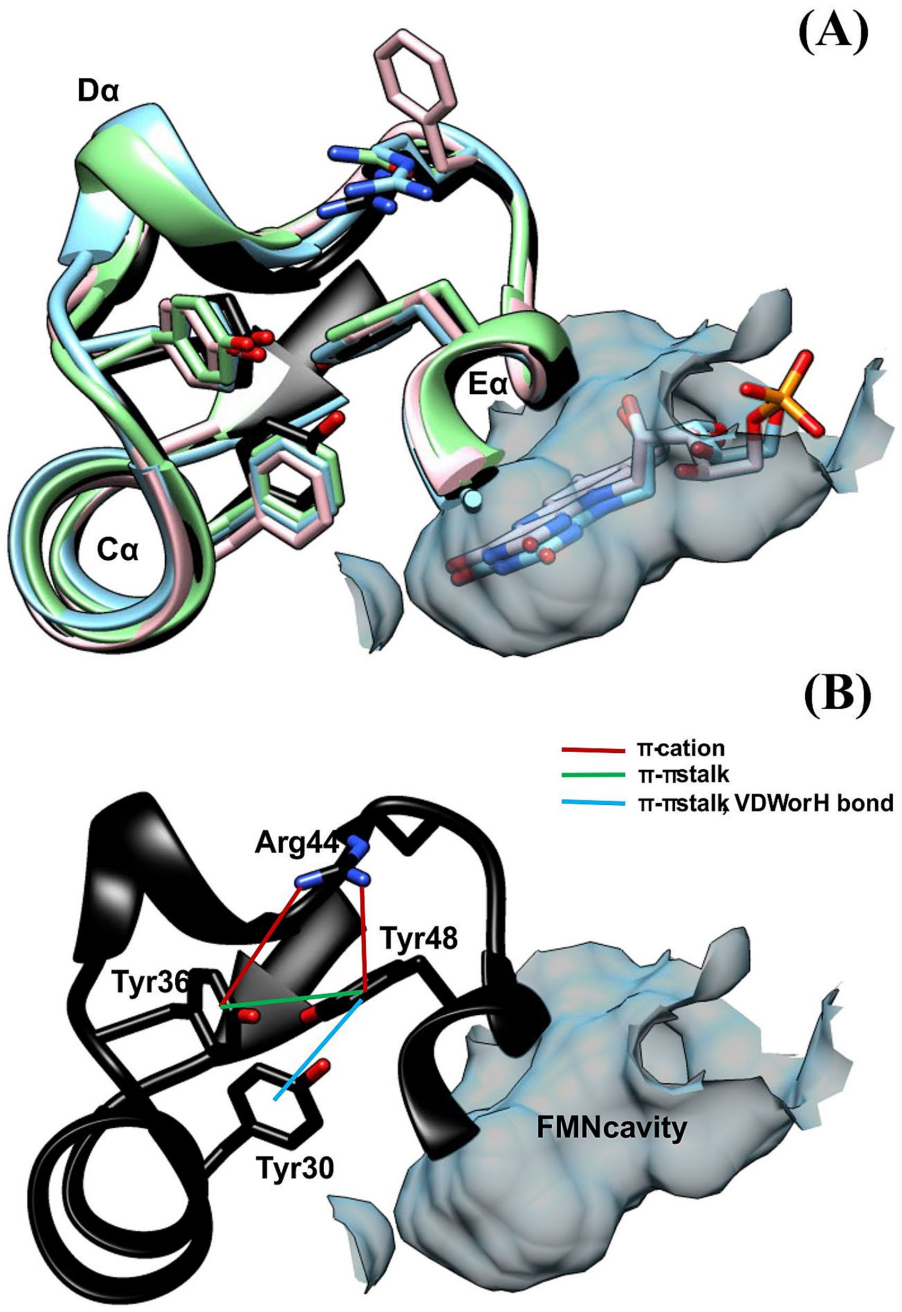
**(A)** Aromatic triad (F-Y-F) located in the loop (Cα-Dα), Eα and Cα for several LOV domains: DsLOV 4KUO (light blue); *Rs*LOV 4HJ4 (pink), PpSB2LOV 7A6P (green) and ALovD-1 (black). **(B)** Same triad for ALovD-1 WT (Y36--Y48--Y30) and predicted interactions between residues.

The analysis of the model obtained from Alphafold2 for Y30F and using Ring4.0 predicted that Tyr48 interacts with Tyr30 through van der Waals (VDW) or ππ stalks according to the conformer in the model. If this is the case, it is possible that Tyr30 could not exert its deactivating effect on flavin because of its interaction with Tyr48 (Figure 6). We prepared a Y48F mutant and found a very low Φ_for_ = 0.13, almost a third of the WT protein, with a discrete but significant increase in the A_sh_/A_max_ (0.83), which suggests that in the Y48F mutant, Tyr30 can interact more freely with flavin in the absence of Tyr48. The flavin Φ_F_ is 0.07, which is half of that for the WT. In this case, the fluorescence decay could only be fitted with a biexponential function, such as in the case of free FMN in PBS. The short component lifetime was greater than that for the WT, and its fractional contribution exceeded 80%. In contrast, the largest lifetime was significantly shorter than that of the WT, rendering an average lifetime that is a fourth of that obtained for the WT at 500 nm.

These results suggest that Tyr30 is an efficient quencher of flavin, but only in the absence of Tyr48, facts that will be discussed later.

## 4 Discussion

Environments such as Lake Diamante are characterized as polyextreme, presenting multiple external factors that hinder microbial survival (Rascovan et al., 2016). The red mat biofilms in this lake are complex communities composed of diverse taxa that collectively function as an organism. Each layer of the biofilms fulfills distinct ecological roles, demonstrating remarkable specialization and adaptation. The archaeal communities within these biofilms endure high intracellular hyperosmolarity, which poses a significant threat to protein stability (Christian and Waltho, 1962; Aharon, 2013). This adaptation is achieved through several strategies that are typical of halo-adapted proteins, including an increased abundance of acidic residues, a higher ratio of arginine to lysine, and an extensive network of salt bridges. Indeed, proteins from halophilic microorganisms present a greater density of negatively charged residues, such as glutamate and aspartate, which reduce solvent contact with the protein surface. This feature minimizes the requirement for water molecules to solvate intracellular salt ions (Paul et al., 2008; Tadeo et al., 2009; Pieper et al., 1998). Our findings on the LOV domains identified in Lake Diamante highlight these adaptive traits. Comparative analyses of three modeled LOV domain sequences (ALovD-1, DL6091, and DL0912) revealed elevated levels of acidic surface residues compared with those in reference LOV domains such as YtvA and VVD. The sum of exposed aspartate and glutamate residues for ALovD-1, DL6091, and DL0912 was 20, 24, and 24, respectively, in contrast to 14 and 21 for VVD and YtvA. Interestingly, YtvA also exhibits traits of halo adaptation, which is consistent with its origin in *Bacillus subtilis*, a microorganism known for having halotolerant proteins (Takenaka et al., 2015; Asha and Sakthivel, 2014; Takenaka et al., 2018). Another halo-adaptation strategy is the reduction in superficial Lys residues and, instead, the presence of Arg. This particular change has been suggested to allow three different types of interactions with surrounding residues on the basis of the asymmetrical distribution of the three nitrogen atoms, in contrast with only one possibility of interaction provided by lysine residues. Additionally, the exchange of Lys for Arg could induce changes in electrostatic interactions in an additional manner, which might be a factor in enhancing stability (Mataga et al., 2002; Borders et al., 1994; Donald and Kulp, 2011). For ALovD-1, DL6091 and DL019, this trend was clear; their surfaces presented Lys ≤ 4.6%, whereas YtvA and VVD accounted for 10 and 8.7%, respectively. On average, more than 10% of the Arg were exposed for the archaeal sequences, whereas less than 7.6% of the residues were exposed for YtvA and VVD.

ALovD-1 was confirmed to be a canonical LOV domain with pronounced halophilic properties and can be classified as a broad-salt-tolerant protein. It retained its activity even at 3 M for monovalent salts with very discrete structural changes. On the basis of the recovery time for thermal relaxation in the dark, it can be cataloged as a slow-cycling LOV (Pudasaini et al., 2015).

Absorption spectroscopy revealed details that were confirmed with the 3D modeled structure of the protein. The UVA vibrational structure is most likely due to the proximity of S14 to both C7M and C8M of ring I from isoalloxazine, similar to what has been described for *As*LOV1 (Salomon et al., 2000). The rotational correlation times of flavin in ALovD-1 are lower than those expected for FMN in its cavity, suggesting that mobility could cause rapid depolarization. However, steady-state anisotropy indicates that the chromophore is quite constrained, especially at high salt concentrations; thus, another process must act, such as charge transfer. This phenomenon has been previously proposed as the formation of a charge transfer complex between flavin in the excited state and Tyr, which causes the rapid depolarization observed (van den Berg et al., 1998).

The exploration of flavin fluorescence and the search for possible quenchers led us to analyze two Tyr residues that are highly conserved as Phe residues in all other LOVs studied thus far and in most of the LOV domains found in Lake Diamante. Single mutants Y30F and Y48F showed that the absence of Y30 did not considerably increase the fluorescence quantum yield of FMN. Instead, the Y48F mutant showed, to a great extent, how Y30 is capable of quenching FMN fluorescence as long as Y48 is not present. To find a suitable explanation for this, as we discussed before, analysis of the 3D model obtained for ALovD-1 highlighted that these two residues may be involved at the same time in the photocycle and in structural stability. Y30 and Y48 in ALovD-1 are Phe in all other LOV sequences. Invariably, the analysis of the structures of various LOV domains (i.e., *D. shibae, R. sphaeroides, P. putida* and ALovD-1 model) position these residues together with a highly conserved Tyr and the X position of the LOV motif (Arinkin et al., 2021; Conrad et al., 2013; Endres et al., 2015). Figure 6A depicts this aromatic triad, in which the already characterized domains is invariably Phe-Tyr-Phe, together with the variable residue of the LOV motif. ALovD-1 presents this triad as Y36-Y48-Y30, which are located in loop Cα-Dα, helices Eα and Cα, respectively (Figure 6B). In ALovD-1, the first two Tyr residues are predicted to interact through *π*π stalk between them and each through a π–cation with R44 (Table 5). Y48 also interacts (according to the model and the rotamer) with Y30 through VDW, ππ stalk or H bond interactions (Supplementary Figure S8). These interactions between Y30 and Y48 may explain why the net effect of Y30 on flavin fluorescence is minimal and why it is released to interact with FMN when Y48 is mutated. Interestingly, the Y30F_446_ mutant was stable at low salt concentrations (0.5 M), whereas the Y48F_446_ fluorescence emission structure was lost within a few hours, and the maximum of emission redshifted to values similar to those of free FMN, suggesting unfolding of the protein (Elcock and McCammon, 1998) and release of the chromophore. When the protein mixture was buffer-exchanged to 3 M KCl, the emission recovered structure, and a hipsochromic effect was observed and not lost when the mutant was kept at 3 M. This effect suggests that Y48F is stable under these conditions and that the chromophore is not released, confirming that the protein structure is unstable at low salt concentrations, allowing the entrance of solvent molecules and the release of flavin, as evidenced by the increase in fluorescence intensity and the batochromic shift in the emission maximum (Supplementary Figure S9). The Y30F mutant did not present this effect. Mutations on a surface residue might be more deleterious for protein stability than those in the inner core, in our case an aromatic residue for another. While Y30 is completely shielded from the solvent, Y48 accounts for 12.1% of the solvent accessible surface area. If Y48 is replaced by a Phe, the exposure decreases to 7.8%. Thus, we propose that Y48 in this archaeal LOV plays two roles: on the one hand, it neutralizes the deactivating ability of Y30 over FMN, not affecting FMN photophysics, which would be equal to having two Phe in the triad, as is the case for all other LOVs. However, Y48 also seems to play a role in structural stabilization when the salt concentration is less than 1 M, as evidenced by flavin fluorescence and described previously. As we have shown, when the salt concentration is increased, the second derivative of the UV absorption spectra suggests that these Tyr and Trp residues are involved in π-cation interactions and are shielded from the solvent toward the hydrophobic core of the protein. Using intrinsic fluorescence, we showed that W87 shields from the solvent at high salt concentrations. All these structural observations suggest that the protein reacts to the high ionic strength of the media to conserve its functionality.

**Table 5:** Predicted interactions of Y48/F48 in ALovD-1 WT and the mutants Y30F and Y48F.

	NodeId1	Interaction	NodeId2	Distance	Donor	Cation
ALovD-1 WT	A:30:_: TYR	VDW: SC_SC	A:48:_: TYR	3.238		
	A:36:_: TYR	PIPISTACK: SC_SC	A:48:_: TYR	4.932		
	A:44:_: ARG	PICATION: SC_SC	A:48:_: TYR	4.242		A:44:_: ARG
	A:44:_: ARG	VDW: SC_SC	A:48:_: TYR	3.235		
Y30F	A:36:_: TYR	PIPISTACK: SC_SC	A:48:_: TYR	5.025		
	A:44:_: ARG	PICATION: SC_SC	A:48:_: TYR	4.038		A:44:_: ARG
Y48F	A:36:_: TYR	PIPISTACK: SC_SC	A:48:_: PHE	4.944		
	A:36:_: TYR	VDW: SC_SC	A:48:_: PHE	2.884		
	A:44:_: ARG	PICATION: SC_SC	A:48:_: PHE	4.346		A:44:_: ARG
	A:44:_: ARG	VDW: SC_SC	A:48:_: PHE	2.757		
	A:48:_: ASN	HBOND: MC_MC	A:48:_: PHE	3.210	A:48:_: PHE	
	A:48:_: ASN	VDW: MC_SC	A:48:_: PHE	2.914		

VDW, van der Waals; SC, side chain; MC, main chain.

In summary, ALovD-1 demonstrates remarkable adaptability to high ionic strength, conserving photocycling functionality even under extreme salinity. The observed adaptations, including increased salt bridges and optimized residue compositions, are a patent proof of its evolution as a robust halophilic photoreceptor. Future research will focus on elucidating the structural determinants underlying its stability and activity in high-salt environments.

## Data Availability

The datasets presented in this study can be found in online repositories. The names of the repository/repositories and accession number(s) can be found in the article/Supplementary material.
