# Supplementary material for: Archaeal LOV domains from Lake Diamante: first functional characterization of a halo-adapted photoreceptor
Source: Front Microbiol. 2025 Jun 13;16:1572269. doi: 10.3389/fmicb.2025.1572269 (PMC12202551; doi:10.3389/fmicb.2025.1572269)
Supplement: Supplementary file 10 [file Table_1.DOCX]

**Table S1:** Full BLAST results for sequences containing LOV domains from Diamante Lake

| **A** | **B** | **C** | **D** | **E** | **F** | **G** | **H** | **I** | **J** | **K** | **L** | **M** | **Specie** | **Family** | **Order** | **Class** | **Phylum** |  |  |  |  |  |  |
| --- | --- | --- | --- | --- | --- | --- | --- | --- | --- | --- | --- | --- | --- | --- | --- | --- | --- | --- | --- | --- | --- | --- | --- |
| **Ga0151614_1001628** | ERH10939.1 | 69.173 | 665 | 199 | 4 | 1 | 661 | 4 | 666 | 0.0 | 902 | 80.00 | *halophilic archaeon J07HX64* | haloarchaea |  | Halobacteria | Euryarchaeotas |  |  |  |  |  |  |
| **Ga0151614_100435** | MCQ4332159.1 | 66.722 | 607 | 147 | 3 | 1 | 561 | 1 | 598 | 0.0 | 791 | 76.44 | *Natronomonas sp. F2-12* | halobacteriaceae | halobacteriales | Halobacteria | Euryarchaeotas |  |  |  |  |  |  |
| **Ga0151614_102304** | MBP1923895.1 | 93.817 | 469 | 29 | 0 | 1 | 469 | 1 | 469 | 0.0 | 895 | 96.59 | *Halorubrum alkaliphilum* | halorubraceae | haloferacales | Halobacteria | Euryarchaeotas |  |  |  |  |  |  |
| **Ga0151614_102516** | CCQ35503.1 | 76.558 | 674 | 156 | 2 | 1 | 673 | 1 | 673 | 0.0 | 1018 | 86.94 | *Natronomonas moolapensis 8.8.11* | halobacteriaceae | halobacteriales | Halobacteria | Euryarchaeotas |  |  |  |  |  |  |
| **Ga0151614_103558** | CCQ36406.1 | 67.203 | 497 | 163 | 0 | 14 | 510 | 142 | 638 | 0.0 | 615 | 78.07 | *Natronomonas moolapensis 8.8.11* | halobacteriaceae | halobacteriales | Halobacteria | Euryarchaeotas |  |  |  |  |  |  |
| **Ga0151614_104473** | MBP1922255.1 | 86.482 | 614 | 83 | 0 | 1 | 614 | 1 | 614 | 0.0 | 1074 | 92.02 | *Halorubrum alkaliphilum* | halorubraceae | haloferacales | Halobacteria | Euryarchaeotas |  |  |  |  |  |  |
| **Ga0151614_104652** | CCQ35503.1 | 76.374 | 673 | 158 | 1 | 1 | 672 | 1 | 673 | 0.0 | 1028 | 86.92 | *Natronomonas moolapensis 8.8.11* | halobacteriaceae | halobacteriales | Halobacteria | Euryarchaeotas |  |  |  |  |  |  |
| **Ga0151614_105846** | SEO10489.1 | 44.654 | 477 | 259 | 3 | 184 | 659 | 255 | 727 | 1.97e-122 | 386 | 62.05 | *Halorientalis persicus* | haloarculaceae | halobacteriales | Halobacteria | Euryarchaeotas |  |  |  |  |  |  |
| **Ga0151614_106091** | QAU14528.1 | 72.293 | 628 | 155 | 7 | 233 | 851 | 352 | 969 | 0.0 | 845 | 80.57 | *Halorubrum sp. BOL3-1* | halorubraceae | haloferacales | Halobacteria | Euryarchaeotas |  |  |  |  |  |  |
| **Ga0151614_107813** | MXR40423.1 | 72.886 | 402 | 109 | 0 | 11 | 412 | 11 | 412 | 0.0 | 621 | 86.57 | *Halobaculum saliterrae* | halorubraceae | haloferacales | Halobacteria | Euryarchaeotas |  |  |  |  |  |  |
| **Ga0151614_110912** | MCL9817540.1 | 69.204 | 578 | 178 | 0 | 1 | 578 | 1 | 578 | 0.0 | 846 | 83.39 | *Natronocalculus amylovorans* | halorubraceae | haloferacales | Halobacteria | Euryarchaeotas |  |  |  |  |  |  |
| **Ga0151614_115602** | OYR46193.1 | 69.020 | 510 | 138 | 6 | 4 | 497 | 16 | 521 | 0.0 | 688 | 79.61 | *Halorubrum sp. Eb13* | halorubraceae | haloferacales | Halobacteria | Euryarchaeotas |  |  |  |  |  |  |
| **Ga0151614_115812** | SIS18214.1 | 47.577 | 454 | 224 | 7 | 1 | 449 | 284 | 728 | 1.89e-125 | 387 | 63.22 | *Natronorubrum thiooxidans* | natrialbaceae | haloferacales | Halobacteria | Euryarchaeotas |  |  |  |  |  |  |
| **Ga0151614_121681** | OYR39992.1 | 81.709 | 667 | 117 | 2 | 1 | 666 | 5 | 667 | 0.0 | 1099 | 89.51 | *Halorubrum sp. Hd13* | halorubraceae | haloferacales | Halobacteria | Euryarchaeotas |  |  |  |  |  |  |
| **Ga0151614_128332** | EMA66818.1 | 69.146 | 363 | 101 | 3 | 1 | 354 | 1 | 361 | 1.38e-171 | 494 | 79.61 | *Halorubrum aidingense JCM 13560* | halorubraceae | haloferacales | Halobacteria | Euryarchaeotas |  |  |  |  |  |  |
| **Ga0151614_143841** | QDX39520.1 | 32.072 | 555 | 324 | 10 | 30 | 540 | 634 | 1179 | 2.36e-83 | 290 | 52.07 | *Salarchaeum sp. JOR-1* | halobacteriaceae | halobacteriales | Halobacteria | Euryarchaeotas |  |  |  |  |  |  |
| **Ga0151614_148921** | MXV61616.1 | 54.034 | 533 | 245 | 0 | 1 | 533 | 177 | 709 | 0.0 | 624 | 75.80 | *Natronorubrum halalkaliphilum* | natrialbaceae | natrialbales | Halobacteria | Euryarchaeotas |  |  |  |  |  |  |
| **Ga0151614_149972** | AUX08044.1 | 69.951 | 406 | 120 | 1 | 49 | 454 | 716 | 1119 | 0.0 | 558 | 81.03 | *Halalkaliarchaeum desulfuricum* | halorubraceae | haloferacales | Halobacteria | Euryarchaeotas |  |  |  |  |  |  |
| **Ga0151614_151402** | MBX0302787.1 | 65.789 | 228 | 77 | 1 | 1 | 227 | 528 | 755 | 2.83e-98 | 308 | 79.39 | *Halomicroarcula salinisoli* | haloarculaceae | halobacteriales | Halobacteria | Euryarchaeotas |  |  |  |  |  |  |
| **Ga0151614_154601** | SDY91998.1 | 67.195 | 442 | 145 | 0 | 3 | 444 | 7 | 448 | 0.0 | 653 | 83.71 | *Halopenitus persicus* | halorubraceae | haloferacales | Halobacteria | Euryarchaeotas |  |  |  |  |  |  |
| **Ga0151614_159301** | TKX80159.1 | 82.783 | 424 | 72 | 1 | 1 | 423 | 42 | 465 | 0.0 | 714 | 91.04 | *Halorubrum sp. SD626R* | halorubraceae | haloferacales | Halobacteria | Euryarchaeotas |  |  |  |  |  |  |
| **Ga0151614_173151** | PHQ40447.1 | 47.475 | 396 | 180 | 5 | 4 | 395 | 226 | 597 | 3.14e-115 | 354 | 65.91 | *Halorubrum persicum* | halorubraceae | haloferacales | Halobacteria | Euryarchaeotas |  |  |  |  |  |  |
| **Ga0151614_178101** | TQQ81405.1 | 69.211 | 380 | 117 | 0 | 3 | 382 | 118 | 497 | 0.0 | 566 | 84.21 | *Halonotius roseus* | halorubraceae | haloferacales | Halobacteria | Euryarchaeotas |  |  |  |  |  |  |
| **Ga0151614_181511** | AUX09055.1 | 56.410 | 156 | 67 | 1 | 12 | 166 | 197 | 352 | 5.52e-48 | 171 | 71.15 | *Halalkaliarchaeum desulfuricum* | halorubraceae | haloferacales | Halobacteria | Euryarchaeotas |  |  |  |  |  |  |
| **Ga0151614_183521** | EMA57890.1 | 78.299 | 341 | 72 | 2 | 2 | 340 | 280 | 620 | 0.0 | 533 | 86.80 | *Halorubrum lipolyticum DSM 21995* | halorubraceae | haloferacales | Halobacteria | Euryarchaeotas |  |  |  |  |  |  |
| **Ga0151614_185661** | RJX44793.1 | 84.488 | 361 | 55 | 1 | 1 | 360 | 72 | 432 | 0.0 | 624 | 89.75 | *Halonotius aquaticus* | halorubraceae | haloferacales | Halobacteria | Euryarchaeotas |  |  |  |  |  |  |
| **Ga0151614_189351** | MCQ4332159.1 | 71.633 | 349 | 92 | 1 | 1 | 349 | 124 | 465 | 6.98e-177 | 509 | 83.67 | *Natronomonas sp. F2-12* | halobacteriaceae | halobacteriales | Halobacteria | Euryarchaeotas |  |  |  |  |  |  |
| **Ga0151614_194791** | QGX93785.1 | 76.190 | 336 | 80 | 0 | 1 | 336 | 112 | 447 | 0.0 | 538 | 89.58 | *Haloplanus rallus* | haloferaceae | haloferacales | Halobacteria | Euryarchaeotas |  |  |  |  |  |  |
| *# Fields: A: query accession ver, B: subject accession ver, C: % identity, D: alignment length, E: mismatches, F: gap opens, G: query start, H: query end,* | | | | | | | | | | | | | | | | | | |  |  | |  |  |
| *I: subject start, J: subject end, K: evalue, L: bit score, M: % positives* | | | | | | | | | | | | | | | | | | | | |  |  |  |

|  |  |  |  |  |  |
| --- | --- | --- | --- | --- | --- |
