# Supplementary material for: Archaeal LOV domains from Lake Diamante: first functional characterization of a halo-adapted photoreceptor
Source: Front Microbiol. 2025 Jun 13;16:1572269. doi: 10.3389/fmicb.2025.1572269 (PMC12202551; doi:10.3389/fmicb.2025.1572269)
Supplement: Supplementary file 11 [file Table_2.DOCX]

|  |  |  |  |  |  |
| --- | --- | --- | --- | --- | --- |

**Table S2:** Gene product names for LOV domain containing ORFs in Diamante Lake Red biofilms metagenome

| Gene ID | Gene Product Name |
| --- | --- |
| **3300011121 assembled Ga0151614_178101** | Bacteriophytochrome (light-regulated signal transduction histidine kinase)/GAF domain-containing protein |
| **3300011121 assembled Ga0151614_102516** | hypothetical protein |
| **3300011121 assembled Ga0151614_104652** | hypothetical protein |
| **3300011121 assembled Ga0151614_102304** | PAS domain-containing protein |
| **3300011121 assembled Ga0151614_143841** | PAS domain-containing protein |
| **3300011121 assembled Ga0151614_128332** | PAS domain-containing protein |
| **3300011121 assembled Ga0151614_181511** | PAS domain-containing protein |
| **3300011121 assembled Ga0151614_189351** | PAS domain-containing protein/Bacteriophytochrome (light-regulated signal transduction histidine kinase) |
| **3300011121 assembled Ga0151614_159301** | PAS domain-containing protein/DNA-binding response regulator, NarL/FixJ family, contains REC and HTH domains |
| **3300011121 assembled Ga0151614_148921** | PAS domain-containing protein/K+-sensing histidine kinase KdpD |
| **3300011121 assembled Ga0151614_151402** | PAS domain-containing protein/PAS domain-containing protein |
| **3300011121 assembled Ga0151614_107813** | PAS domain-containing protein/PAS domain-containing protein |
| **3300011121 assembled Ga0151614_154601** | PAS domain-containing protein/PAS domain-containing protein |
| **3300011121 assembled Ga0151614_115812** | PAS domain-containing protein/PAS domain-containing protein/K+-sensing histidine kinase KdpD |
| **3300011121 assembled Ga0151614_194791** | PAS domain-containing protein/PAS domain-containing protein/PAS domain-containing protein |
| **3300011121 assembled Ga0151614_185661** | PAS domain-containing protein/PAS domain-containing protein/PAS domain-containing protein |
| **3300011121 assembled Ga0151614_103558** | PAS domain-containing protein/Signal transduction histidine kinase |
| **3300011121 assembled Ga0151614_100435** | PAS domain-containing protein/Signal transduction histidine kinase/DNA-binding transcriptional response regulator, NtrC family, contains REC, AAA-type ATPase, and a Fis-type DNA-binding domains/Bacteriophytochrome (light-regulated signal transduction histidine kinase) |
| **3300011121 assembled Ga0151614_106091** | PAS domain-containing protein/Signal transduction histidine kinase/PAS domain-containing protein |
| **3300011121 assembled Ga0151614_121681** | Predicted DNA binding protein, contains HTH domain/PAS domain-containing protein |
| **3300011121 assembled Ga0151614_1001628** | Predicted DNA binding protein, contains HTH domain/PAS domain-containing protein |
| **3300011121 assembled Ga0151614_173151** | Signal transduction histidine kinase |
| **3300011121 assembled Ga0151614_183521** | Signal transduction histidine kinase |
| **3300011121 assembled Ga0151614_110912** | Signal transduction histidine kinase |
| **3300011121 assembled Ga0151614_115602** | Signal transduction histidine kinase |
| **3300011121 assembled Ga0151614_104473** | Signal transduction histidine kinase/DNA-binding transcriptional response regulator, NtrC family, contains REC, AAA-type ATPase, and a Fis-type DNA-binding domains |
| **3300011121 assembled Ga0151614_149972** | Signal transduction histidine kinase/PAS domain-containing protein |
| **3300011121 assembled Ga0151614_105846** | Signal transduction histidine kinase/PAS domain-containing protein/PAS domain-containing protein |
