# Supplementary material for: Archaeal LOV domains from Lake Diamante: first functional characterization of a halo-adapted photoreceptor
Source: Front Microbiol. 2025 Jun 13;16:1572269. doi: 10.3389/fmicb.2025.1572269 (PMC12202551; doi:10.3389/fmicb.2025.1572269)
Supplement: Supplementary file 12 [file Table_3.DOCX]

**Table S3:** Salt bridges as calculated via RING4.0 for ALovD-1, DL6091 and DL0912 (modeled PDBs), YtvA (2MWG) and VVD (3RH8). To make comparable calculations, each PDB was modified, and only 120 residues from the LOV core were left, excluding A´α and most of the J α in all structures.

| **ALovD-1** |  |  |  |  |
| --- | --- | --- | --- | --- |
| **NodeId1** | **NodeId2** | **Distance** | **Angle** | **Positive** |
| A:20:_:ASP^a,b^ | A:47:_:ARG | 3.546 | 105.699 | A:47:_:ARG |
| A:28:_:ASP^a,b^ | A:38:_:LYS | 2.733 | 72.461 | A:38:_:LYS |
| A:40:_:GLU^a,b^ | A:81:_:LYS | 2.660 | 101.752 | A:81:_:LYS |
| A:61:_:GLU^b^ | A:64:_:LYS | 2.800 | 33.545 | A:64:_:LYS |
| A:64:_:LYS^b^ | A:70:_:GLU | 3.087 | 73.974 | A:64:_:LYS |
| A:69:_:GLU^b^ | A:96:_:ARG | 3.402 | 78.996 | A:96:_:ARG |
| A:77:_:ARG^b^ | A:114:_:GLU | 3.476 | 96.902 | A:77:_:ARG |
| A:75:_:GLU^a^ | A:115:_:ARG | 3.896 | 114.687 | A:118:_:ARG |
| A:89:_:ARG^a,b^ | A:110:_:GLU | 3.802 | 26.511 | A:89:_:ARG |
| A:116:_:LYS^b^ | A:119:_:GLU | 2.708 | 47.971 | A:116:_:LYS |
| **YtvA** |  |  |  |  |
| A:85:_:LYS | A:112:_:GLU | 3.674 | 106.325 | A:85:_:LYS |
| **VVD** |  |  |  |  |
| A:57:_:ARG | A:144:_:GLU | 3.720 | 128.846 | A:57:_:ARG |
| A:77:_:ASP | A:176:_:ARG | 3.739 | 43.411 | A:176:_:ARG |
| A:82:_:ASP | A:109:_:ARG | 3.701 | 114.064 | A:109:_:ARG |
| A:102:_:GLU | A:154:_:LYS | 3.117 | 89.045 | A:154:_:LYS |
| A:153:_:LYS | A:184:_:GLU | 3.297 | 99.762 | A:153:_:LYS |
| **DL6091** |  |  |  |  |
| A:33:_:ASP | A:60:_:ARG | 3.491 | 115.648 | A:60:_:ARG |
| A:41:_:ASP | A:51:_:ARG | 3.531 | 67.845 | A:51:_:ARG |
| A:45:_:GLU | A:93:_:ARG | 3.668 | 77.374 | A:93:_:ARG |
| A:53:_:GLU | A:94:_:LYS | 2.775 | 137.192 | A:94:_:LYS |
| A:70:_:GLU | A:73:_:LYS | 3.061 | 28.548 | A:73:_:LYS |
| A:73:_:LYS | A:77:_:GLU | 2.803 | 46.382 | A:73:_:LYS |
| A:76:_:ARG | A:80:_:ASP | 3.498 | 61.206 | A:76:_:ARG |
| A:82:_:GLU | A:109:_:ARG | 3.375 | 120.324 | A:109:_:ARG |
| A:88:_:GLU | A:129:_:ARG | 3.384 | 69.874 | A:129:_:ARG |
| A:90:_:ARG | A:98:_:GLU | 3.344 | 62.559 | A:90:_:ARG |
| **DL0912** |  |  |  |  |
| A:41:_:GLU | A:83:_:ARG | 3.419 | 120.759 | A:83:_:ARG |
| A:48:_:ASP | A:58:_:ARG | 3.470 | 75.557 | A:58:_:ARG |
| A:60:_:GLU | A:101:_:LYS | 2.626 | 105.126 | A:101:_:LYS |
| A:77:_:GLU | A:81:_:ARG | 3.503 | 39.886 | A:81:_:ARG |
| A:95:_:GLU | A:136:_:LYS | 3.788 | 91.687 | A:136:_:LYS |
| A:130:_:GLU | A:136:_:LYS | 3.719 | 103.949 | A:136:_:LYS |
