# Supplementary material for: Archaeal LOV domains from Lake Diamante: first functional characterization of a halo-adapted photoreceptor
Source: Front Microbiol. 2025 Jun 13;16:1572269. doi: 10.3389/fmicb.2025.1572269 (PMC12202551; doi:10.3389/fmicb.2025.1572269)
Supplement: Supplementary file 13 [file Table_4.DOCX]

**Table S4.** Surface exposed residues (≥10%) for ALovD-1, DL-6091 and DL-0912 (modeled PDBs), YtvA (PDB: 2MWG) and VVD (PDB:3RH8) were calculated via SwissPDBViewer.

|  | **ALovD-1** |  | **DL6091** |  | **DL0912** |  | **YtvA** |  | **VVD** |  |
| --- | --- | --- | --- | --- | --- | --- | --- | --- | --- | --- |
| **Residue** | **Number** | **%** | **Number** | **%** | **Number** | **%** | **Number** | **%** | **Number** | **%** |
| **Ala (A)** | 5 | 5.7 | 3 | 3.5 | 5 | 5.6 | 2 | 2.2 | 4 | 4.3 |
| **Arg (R)** | 9 | 10.3 | 10 | 11.6 | 12 | 13.3 | 2 | 2.2 | 7 | 7.6 |
| **Asn (N)** | 3 | 3.4 | 6 | 7.0 | 1 | 1.1 | 6 | 6.7 | 7 | 7.6 |
| **Asp (D)** | 9 | 10.3 | 9 | 10.5 | 12 | 13.3 | 9 | 10.1 | 6 | 6.5 |
| **Cys (C)** | 0 | 0.0 | 1 | 1.2 | 1 | 1.1 | 0 | 0.0 | 1 | 1.1 |
| **Gln (Q)** | 5 | 5.7 | 6 | 7.0 | 2 | 2.2 | 7 | 7.9 | 5 | 5.4 |
| **Glu (E)** | 11 | 12.6 | 15 | 17.4 | 12 | 13.3 | 12 | 13.5 | 8 | 8.7 |
| **Gly (G)** | 8 | 9.2 | 5 | 5.8 | 6 | 6.7 | 5 | 5.6 | 8 | 8.7 |
| **His (H)** | 0 | 0.0 | 0 | 0.0 | 2 | 2.2 | 1 | 1.1 | 0 | 0.0 |
| **Ile (I)** | 5 | 5.7 | 7 | 8.1 | 5 | 5.6 | 3 | 3.4 | 1 | 1.1 |
| **Leu (L)** | 2 | 2.3 | 1 | 1.2 | 3 | 3.3 | 5 | 5.6 | 5 | 5.4 |
| **Lys (K)** | 4 | 4.6 | 3 | 3.5 | 2 | 2.2 | 9 | 10.1 | 8 | 8.7 |
| **Met (M)** | 0 | 0.0 | 1 | 1.2 | 3 | 3.3 | 3 | 3.4 | 3 | 3.3 |
| **Phe (F)** | 2 | 2.3 | 2 | 2.3 | 3 | 3.3 | 2 | 2.2 | 1 | 1.1 |
| **Pro (P)** | 7 | 8.0 | 4 | 4.7 | 3 | 3.3 | 5 | 5.6 | 7 | 7.6 |
| **Ser (S)** | 2 | 2.3 | 1 | 1.2 | 8 | 8.9 | 1 | 1.1 | 3 | 3.3 |
| **Thr (T)** | 7 | 8.0 | 3 | 3.5 | 3 | 3.3 | 6 | 6.7 | 7 | 7.6 |
| **Trp (W)** | 1 | 1.1 | 1 | 1.2 | 1 | 1.1 | 1 | 1.1 | 0 | 0.0 |
| **Tyr (Y)** | 4 | 4.6 | 3 | 3.5 | 2 | 2.2 | 5 | 5.6 | 6 | 6.5 |
| **Val (V)** | 3 | 3.4 | 5 | 5.8 | 4 | 4.4 | 5 | 5.6 | 4 | 4.3 |
