# Supplementary material for: Archaeal LOV domains from Lake Diamante: first functional characterization of a halo-adapted photoreceptor
Source: Front Microbiol. 2025 Jun 13;16:1572269. doi: 10.3389/fmicb.2025.1572269 (PMC12202551; doi:10.3389/fmicb.2025.1572269)
Supplement: Supplementary file 14 [file Table_5.DOCX]

**Table S5:** Solvent Accessible Surface Area as calculated using GetArea (1)

| **Protein** | **Residue** | **Total** | **Apolar** | **Backbone** | **Sidechain** | **Ratio(%)** | **In/Out** |
| --- | --- | --- | --- | --- | --- | --- | --- |
| **WT** | **TYR 30** | 0 | 0 | 0 | 0 | 0 | i |
|  | **TYR 36** | 33.17 | 10.80 | 0.00 | 33.17 | 17.2 | i |
|  | **CYS 46** | 33.76 | 23.66 | 2.12 | 31.64 | 30.9 |  |
|  | **TYR 48** | 50.19 | 35.94 | 26.92 | 23.27 | 12.1 | i |
| **Y48F** | **TYR 30** | 0 | 0 | 0 | 0 | 0 | i |
|  | **TYR 36** | 36.12 | 13.50 | 0.57 | 35.56 | 18.4 | i |
|  | **CYS 46** | 35.36 | 25.93 | 2.50 | 32.86 | 32.1 |  |
|  | **PHE 48** | 41.00 | 26.32 | 26.92 | 14.08 | 7.8 | i |
| **Y30F** | **PHE 30** | 0 | 0 | 0 | 0 | 0 | i |
|  | **TYR 36** | 31.22 | 10.01 | 0.00 | 31.22 | 16.2 | i |
|  | **CYS 46** | 34.54 | 23.66 | 2.67 | 31.87 | 31.2 |  |
|  | **TYR 48** | 46.76 | 33.03 | 25.65 | 21.10 | 10.9 | i |
